# TyG index and CBC-derived inflammatory indicators individual and mixed effects on all-cause and cardiovascular disease deaths in patients with CHD

**DOI:** 10.3389/fcvm.2025.1600097

**Published:** 2025-09-10

**Authors:** Lipeng Cai, Jiangrong Yan, Lei Sun, Weichao Dan

**Affiliations:** 1 Department of Cardiology, The Third People’s Hospital of Huizhou, The Affiliated Hospital of Guangzhou Medical University, Huizhou, Guangdong, China; 2 Cardiovascular Department, Huizhou Affiliated Hospital of Sun Yat-sen University, Huizhou, Guangdong, China; 3 Orthopedic Department, Guoyao North Hospital, Baotou, Inner Mongolia Autonomous Region, China

**Keywords:** TyG index, CBC inflammatory marker, all-cause mortality, cardiovascular mortality, environmental health, CBC-derived inflammatory index, CHD, cardiovascular disease mortality

## Abstract

**Background and aims:**

Currently, most epidemiological investigations have concentrated on exploring the correlation between a singular indicator and the risk of cardiovascular disease. In clinical practice, a single indicator often fails to comprehensively represent a patient's health status. Consequently, it is crucial to thoroughly evaluate the influence of multiple indicators on disease prediction. Recently, the Triglyceride-Glucose Index (TyG Index) and inflammatory markers derived from CBC (CBC) have garnered increasing attention. Nevertheless, research on the individual and synergistic impacts of the TyG index and inflammatory indices on the risk of all-cause mortality and cardiovascular death in patients with coronary heart disease (CHD) remains scarce. To develop a more accurate risk assessment instrument for the clinic and to provide a novel strategy and framework for the management of individuals with coronary artery disease (CAD).

**Methods and results:**

This study identified patients with CHD aged 20 years and older from five cycles of National Health and Nutrition Examination Survey (NHANES) data spanning 2009 to 2018. We employed weighted logistic regression analysis and the restricted cubic spline (RCS) approach to investigate the TyG index and CBC-derived inflammatory indicators in relation to the risk of all-cause mortality and cardiovascular mortality in patients with CHD. The cumulative exposure effect of these measures was estimated utilizing a Weighted Quantitative Scoring (WQS) model. In the unadjusted model, ln(SII) (OR = 1.8, 95% CI = 1.05 ∼ 3.06, *P* = 0.032), ln(SIRI) (OR = 2.08, 95% CI = 1.45 ∼ 2.98, *P* < 0.001), ln(MLR) (OR = 2.53, 95% CI = 1.55 ∼ 4.13, *P* < 0.001), ln(NLR) (OR = 2.57, 95% CI = 1.44 ∼ 4.60, *P* = 0.002), and ln(PLR) (OR = 2.56, 95% CI = 1.53 ∼ 4.29, *P* < 0.001) exhibited a positive correlation with the risk of all-cause mortality. In the model, after comprehensive adjustment for confounders, it continued to exhibit a substantial association with the risk of mortality from CHD. RCS analysis revealed a nonlinear dose-response relationship between Monocyte-to-Lymphocyte Ratio (MLR), Neutrophil-to-Lymphocyte Ratio (NLR), Platelet-to-Lymphocyte Ratio (PLR), Systemic Immune-Inflammation Index (SII), and Systemic Inflammation Response Index (SIRI) and the probability of all-cause mortality (*P* -nonlinear < 0.05). The WQS model indicated that simultaneous exposure to the TyG index and inflammatory markers derived from CBC was significantly and positively associated with the risk of all-cause mortality and cardiovascular death in patients with CAD (OR = 1.68, 95% CI = 1.22–2.30, *P* = 0.009, and OR = 2.2, 95% CI = 1.42–3.42, *P* = 0.0004), with the Neutrophil-to-Platelet Ratio (NPR) and SII being the most significant contributors to the overall risk.

**Conclusion:**

Our investigation demonstrated that the TyG index and inflammatory indices generated from CBC are significant risk factors for all-cause mortality and cardiovascular mortality in patients with CHD, with NPR and SII representing the largest proportion of these factors.

## 1 Introduction

Cardiovascular Diseases (CVD) include disorders of the heart and blood vessels (1), including CHD, coronary artery disease (CAD), and acute coronary syndrome (ACS). CHD originates from CAD, and while the terms “CAD” and “CHD” are frequently used interchangeably, CHD is the final clinical manifestation of CAD (2). CHD has historically been the predominant cause of deaths associated with CVD globally and significantly elevates the risk of all-cause mortality. Despite lifestyle modifications and management of prevalent risk factors (e.g., diabetes mellitus, hypertension, and high cholesterol) being primary strategies to mitigate the risk of cardiovascular and overall mortality from CHD, the occurrence of cardiovascular events remains elevated, even as individuals increasingly focus on health management and effectively diminish the influence of traditional risk factors. Consequently, in recent years, blood glucose, blood lipids, and chronic inflammation markers have emerged as critical determinants warranting significant research and clinical attention. Insulin resistance (IR), an indicator intimately associated with blood glucose and lipids, has been gradually emphasized.

IR, characterized by diminished sensitivity of insulin target tissues to its metabolic effects, serves as a prevalent pathophysiological foundation for metabolic syndrome, obesity, and diabetes, as well as a significant risk factor for cardiovascular disease (3, 4). The TyG index has garnered significant attention in recent years as a novel metric for evaluating IR (5). The index is derived from fasting blood glucose and triglyceride levels. Compared to the conventional high-insulin normoglycemic clamp test, it offers notable advantages, including ease of operation, low cost, and noninvasiveness, which renders it more appropriate for extensive clinical application (6). An increasing amount of research indicates that the TyG index is significantly correlated with the onset and progression of cardiovascular illnesses (7–10). A greater TyG index often indicates excessive blood glucose levels and dyslipidemia, which can exacerbate the progression of cerebral-cardiovascular atherosclerosis, hence heightening the risk of CHD and other cardiovascular conditions.

Inflammation is pivotal in the development and advancement of CVD, particularly in the pathophysiologic pathways of CHD and atherosclerosis. Conventional inflammatory indicators, including C-Reactive Protein (CRP), are extensively utilized in clinical practice. Recently, innovative inflammation indicators derived from CBC (CBC) have gradually garnered attention. These indicators can thoroughly reflect the immunological and inflammatory status of the body and provide potential clinical relevance in the diagnosis, risk assessment, and prognosis of cardiovascular illnesses.

Systemic Immune-Inflammation Index (SII) is an extensive inflammatory marker derived from peripheral blood neutrophil, platelet, and lymphocyte counts. Research indicates that SII is significantly associated with systemic immune response and inflammatory conditions, perhaps contributing to the atherosclerotic process. Furthermore, elevated levels of SII are strongly associated with the initiation and advancement of cardiovascular illness, indicating its significance in cardiovascular disease risk assessment (11). As a key inflammatory marker, the Systemic Inflammation Response Index (SIRI), as a pivotal inflammatory marker, can thoroughly represent the immunological and inflammatory equilibrium of the body by amalgamating neutrophil, monocyte, and lymphocyte numbers (12). Research indicates that elevated SIRI levels are strongly correlated with a substantial rise in CHD risk, implying its possible application in the prognostic evaluation of cardiovascular disease (13). Monocyte-to-Lymphocyte Ratio (MLR) is defined as the ratio of monocyte count to lymphocyte count. A higher MLR was strongly correlated with an elevated risk of Major Adverse Cardiovascular Events (MACE) in patients with CHD, indicating its crucial function in the risk stratification of CHD. The Neutrophil-to-Lymphocyte Ratio (NLR) serves as a significant inflammatory biomarker. An elevation in the number of neutrophils is closely linked to inflammation, whereas a reduction in lymphocyte count correlates with the stress response. Consequently, the NLR reflects the equilibrium between inflammation and immune responses, holding clinical significance in cardiovascular disease. An elevated NLR is associated with a heightened risk of cardiovascular and overall mortality in individuals with CHD. Platelet-to-Lymphocyte Ratio (PLR) is the ratio of platelets to lymphocytes in peripheral circulation. PLR demonstrates superior predictive capability for coronary atherosclerotic burden compared to lymphocyte or platelet counts individually, with studies indicating a significant correlation between PLR and the risk of CHD (14, 15). The Neutrophil-to-Platelet Ratio (NPR) has been examined infrequently in patients with CHD, but there is increasing interest in its prospective therapeutic significance.

Notwithstanding the potential applications of CBC-derived inflammatory indices (e.g., SII, SIRI, MLR, NLR, PLR, and NPR) as well as the TyG index in cardiovascular disease, numerous hurdles persist. The function of these indicators across various populations and disease phases, as well as their specific mechanisms of action, needs further investigation in more studies. Furthermore, integrating these indicators with current clinical assessment techniques to enhance the diagnostic and prognostic evaluation of cardiovascular illnesses represents an important avenue for future research. However, previous studies have primarily examined these markers individually, and limited research has investigated their combined predictive value in CHD patients. Additionally, most studies have not employed advanced statistical methods like weighted quantile sum (WQS) regression to assess the cumulative effect of multiple inflammatory markers simultaneously. Therefore, this study aimed to systematically assess the efficacy of the combined application of the TyG index and CBC-derived inflammation index in patients with CAD, while investigating its potential contributions to disease diagnosis, risk stratification, and prognostic evaluation, thereby offering novel insights and justifications for future precision medicine in cardiovascular disease.

## 2 Methods

### 2.1 Data collection

#### 2.1.1 Database overview

Data from the National Health and Nutrition Examination Survey (NHANES) were obtained for analysis, with approval from the Ethics Review Board of the National Center for Health Statistics. NHANES employs a multistage, stratified, random sampling methodology as a nationally representative cross-sectional survey conducted biennially since 1999 to assess the health and nutritional status of the U.S. population.

#### 2.1.2 Study population included in the database

Data was gathered from 49,693 adults aged 20 years or older with CHD from NHANES 2009–2018 who had information on blood index tests. Individuals were classified as having CHD in this study if they met one of the following criteria: a physician diagnosed them with CHD; a physician diagnosed them with angina pectoris; or a physician informed them that they had experienced a myocardial infarction. Initially, we excluded 20,874 respondents without CHD subjects, followed by 5 individuals with missing death data (either lost to follow-up or under 20 years of age), then 1,157 subjects with in CBCs, and finally 32 participants with absent covariate data. In conclusion, our analysis comprised 793 individuals.

#### 2.1.3 Measuring indicators of inflammation based on blood cell counts

Blood cell counts were obtained using a Beckman Coulter automated hematology analyzer. Inflammatory indices were calculated as follows: SIRI = neutrophil count × (monocyte count/lymphocyte count); SII = platelet count × (neutrophil count/lymphocyte count); MLR = monocyte count/lymphocyte count; NLR = neutrophil count/lymphocyte count; PLR = platelet count/lymphocyte count; NPR = neutrophil count/platelet count. The TyG index was calculated as: ln [fasting triglyceride [mg/dl] × fasting glucose [mg/dl]/2].

#### 2.1.4 All-cause mortality & cardiovascular disease-specific mortality in patients with CHD

To ascertain the cause of death and mortality status until the conclusion of the follow-up period on December 31, 2019, mortality data were linked to the National Death Index Public Access Files. The International Classification of Diseases, Tenth Revision (ICD-10), was employed to categorize the causes of mortality. The principal research outcomes were mortality from cardiovascular disease and overall mortality. Throughout the follow-up period, mortality from any cause was classified as all-cause mortality. Mortality from cardiovascular illness encompasses fatalities resulting from cardiac diseases (ICD-10 codes: I00-I09, I11, I13, I20-I51) and cerebrovascular diseases (ICD-10 codes: I60-I69).

#### 2.1.5 Assessment of other covariates

Demographic data gathered from participants included age, gender (male or female), race/ethnicity (Mexican American, other Hispanic, non-Hispanic White, non-Hispanic Black, or other), and education level (<9th grade; 9th-11th grade, including 12th grade without a diploma; high school graduate/GED or equivalent; some college or an associate's degree; or a college degree or higher). The poverty income ratio (PIR) was calculated by dividing household income by the poverty threshold according to the household's size, year, and state. PIR was categorized into two classifications for analysis: low income (poverty) and middle to high income. Lifestyle factors were extracted from questionnaire replies. The interview question, “Have you smoked at least 100 cigarettes in your lifetime?” was employed to evaluate smoking status. Respondents who answered “No” were categorized as “non-smokers,” whereas those who responded “Yes” were classified as “smokers.” Alcohol consumption was categorized based on the average daily intake of beverages during the prior 12 months. A normal drink is defined as 12 ounces of beer, 1.5 ounces of distilled spirits, or 5 ounces of wine. Participants were classified into four categories: fewer than 5 drinks per day, 5–10 drinks per day, more than 10 drinks per day, and ambiguous alcohol intake. Physical activity was categorized according to participants' responses regarding their engagement in vigorous physical activity, which was defined as any exercise, fitness regimen, or recreational pursuit that significantly elevates respiration or heart rate, including jogging or basketball, sustained for at least 10 min, or moderate recreational activities, which encompass exercises that cause slight elevations in respiration or heart rate, such as brisk walking, cycling, volleyball, or swimming, also for a minimum duration of 10 min. Participants were classified as physically active if they reported engaging in any activity at least once per week and as inactive if they did so less than once per week. Body mass index (BMI, kg/m^2^) was calculated by dividing weight (kg) by the square of height (m^2^). Hypertension was categorized based on self-reported information, specifically if a physician or healthcare professional had ever diagnosed the participant with elevated blood pressure. Hypercholesterolemia was characterized by the prior notification of increased cholesterol values to the participant by a physician or other healthcare provider.

### 2.2 Statistical analysis methods

R (v4.4.0) was employed for all statistical analyses. The Shapiro–Wilk test was utilized to confirm the normality of the continuous data. If the distribution was non-normal, the data will be described with the median and interquartile range (IQR), whereas normally distributed data is represented by frequency and mean ± standard deviation (SD). Counts and percentages were adopted to represent categorical variables. Multifactorial logistic regression models were employed to calculate odds ratios (ORs) and 95% confidence intervals (CIs) regarding the relationship between blood inflammation indices and the TyG index with the risk of all-cause and cardiovascular mortality in patients with CHD, as well as to evaluate the associations of individual blood inflammation indices and the TyG index with the risk of all-cause and cardiovascular mortality in this patient population. Three hierarchical multivariable models were constructed:

Model 1: Unadjusted analysis.

Model 2: Adjusted for demographic factors (age, gender, race, education, household income-to-poverty ratio) and BMI.

Model 3: Additionally adjusted for lifestyle and clinical factors (smoking status, alcohol consumption, hypertension, diabetes, cholesterol levels, physical activity).

Model 1 was unadjusted for covariates; model 2 was adjusted from model 1 for gender, age, BMI, race, education, and household income-to-poverty ratio; and model 3 was adjusted on the basis of model 2 for smoking status, alcohol consumption, hypertension, cholesterol levels, diabetes presence, and physical activity. The dose-response relationships between blood inflammation indices and the TyG index concerning the risk of all-cause and cardiovascular mortality in patients with CHD were evaluated using restricted cubic spline bars. Weighted Quantitative Scoring (WQS) regressions were employed to investigate the association between the aggregated exposure to blood inflammation indices and the TyG index with all-cause and cardiovascular mortality related to CHD. The cumulative impact of median alterations across all indices was subsequently analyzed, and the proportional contribution (weights) of each indicator was determined. Two-sided *P* values less than 0.05 were deemed statistically significant.

## 3 Results

### 3.1 Baseline characteristics of participants

Table 1 delineates the characteristics of individuals with CAD categorized by all-cause mortality classification; (Table 2 presents the characteristics of the sample stratified by cardiovascular disease deaths). This study comprised a total of 793 patients with CHD.

**Table 1:** Population characteristics of all-cause death research, NHANES 2011–2018.

Characteristic	*N* ^a^	Overall *N* = 5,764,7272	0, *N* = 588	1, *N* = 205	*p*-value
Avg # alcohol drinks/day - past 12 mos	793				0.017
Drink heavily (>10)		7 (1.1%)	5 (1.2%)	2 (0.6%)	
Drink a little. (1–5)		381 (55%)	303 (59%)	78 (40%)	
Moderate drinking (6–10)		18 (3.1%)	13 (3.1%)	5 (3.1%)	
Unknow		387 (41%)	267 (37%)	120 (56%)	
Gender	793				0.835
Women		312 (40%)	240 (40%)	72 (41%)	
Men		481 (60%)	348 (60%)	133 (59%)	
Race	793				0.535
Mexican American		66 (3.7%)	51 (3.8%)	15 (3.2%)	
Non-Hispanic Black		138 (8.0%)	109 (8.4%)	29 (6.3%)	
Non-Hispanic White		452 (78%)	315 (77%)	137 (81%)	
Other Hispanic		78 (4.4%)	64 (4.8%)	14 (2.8%)	
Other race - including multi-racial		59 (6.1%)	49 (6.0%)	10 (6.6%)	
Education	793				0.299
9–11th grade (includes 12th grade with no diploma)		124 (13%)	88 (12%)	36 (15%)	
College graduate or above		141 (23%)	111 (24%)	30 (19%)	
High school graduate/GED or equivalent		191 (26%)	138 (26%)	53 (26%)	
Less than 9th grade		122 (9.5%)	84 (8.1%)	38 (14%)	
Some college or AA degree		215 (29%)	167 (30%)	48 (26%)	
Ratio of family income to poverty	793	2.47 (1.29, 4.25)	2.65 (1.38, 4.64)	1.90 (1.24, 3.06)	0.002
Vigorous recreational activities	793	58 (8.5%)	53 (10%)	5 (2.1%)	<0.001
Moderate recreational activities	793	262 (38%)	215 (42%)	47 (23%)	<0.001
Smoked at least 100 cigarettes in life	793	475 (62%)	351 (62%)	124 (64%)	0.63
Diabetes	793	356 (40%)	249 (38%)	107 (48%)	0.044
Body mass index (kg/m**2)	793	29 (25, 34)	29 (26, 34)	30 (25, 34)	0.369
Hypertension	793	602 (73%)	439 (72%)	163 (77%)	0.356
High cholesterol	793	540 (70%)	401 (70%)	139(69%)	0.875
Age (years)	793	66 (58, 76)	65 (56, 72)	75 (66, 80)	<0.001

Any covariate does not adjust model 1; Model 2 adjusts gender, age, body mass index, race, education level, family income and poverty ratio on the basis of model 1; Model 3 adjusted smoking status, drinking status, hypertension, cholesterol, diabates and physical activity on the basis of model 2.

a *N* not missing (unweighted).

b *n* (unweighted) (%); median (Q1, Q3).

c Pearson's *X*^2^: Rao & Scott adjustment; design-based KruskalWallis test.

**Table 2:** Population characteristics of death from cardiovascular and cerebrovascular diseases., NHANES 2009–2018.

Characteristic	*N* ^a^	Overall *N* = 793	0, *N* = 588	1, *N* = 205	*p*-value^c^
Avg # alcohol drinks/day - past 12 mos	793				<0.001
Drink heavily (>10)		7 (1.1%)	6 (1.1%)	1 (1.0%)	
Drink a little. (1–5)		381 (55%)	354 (56%)	27 (35%)	
Moderate drinking (6–10)		18 (3.1%)	17 (3.3%)	1 (0.8%)	
Unknow		387 (41%)	335 (39%)	52 (64%)	
Gender	793				0.092
Men		312 (40%)	291 (41%)	21 (31%)	
Women		481 (60%)	421 (59%)	60 (69%)	
Race	793				0.912
Mexican American		66 (3.7%)	62 (3.8%)	4 (2.9%)	
Non-Hispanic Black		138 (8.0%)	125 (8.0%)	13 (7.2%)	
Non-Hispanic White		452 (78%)	400 (78%)	52 (78%)	
Other Hispanic		78 (4.4%)	71 (4.4%)	7 (4.1%)	
Other race - including multi-racial		59 (6.1%)	54 (6.0%)	5 (7.6%)	
Education	793				0.474
9–11th grade (includes 12th grade with no diploma)		124 (13%)	110 (13%)	14 (12%)	
College graduate or above		141 (23%)	124 (22%)	17 (29%)	
High school graduate/GED or equivalent		191 (26%)	172 (26%)	19 (27%)	
Less than 9th grade		122 (9.5%)	108 (9.3%)	14 (12%)	
Some college or AA degree		215 (29%)	198 (30%)	17 (20%)	
Ratio of family income to poverty	793	2.47 (1.29, 4.25)	2.47 (1.29, 4.25)	2.06 (1.39, 4.84)	0.882
Vigorous recreational activities	793	58 (8.5%)	55 (8.8%)	3 (4.2%)	0.203
Moderate recreational activities	793	262 (38%)	239 (38%)	23 (33%)	0.269
Smoked at least 100 cigarettes in life	793	475 (62%)	436 (63%)	39 (48%)	0.041
diabetes	793	356 (40%)	314 (39%)	42 (48%)	0.183
Body mass index (kg/m*2)	793	29 (25, 34)	29 (25, 34)	30 (25, 34)	0.811
Hypertension	793	602 (73%)	539 (73%)	63 (73%)	0.938
High cholesterol	793	540 (70%)	484 (70%)	56(69%)	0.902
Age (years)	793	66 (58, 76)	66 (57, 74)	76 (69, 80)	<0.001

Any covariate does not adjust model 1; Model 2 adjusts gender, age, body mass index, race, education level, family income and poverty ratio on the basis of model 1; Model 3 adjusted smoking status, drinking status, hypertension, cholesterol, diabetes and physical activity on the basis of model 2.

a *N* not missing (unweighted).

b *n* (unweighted) (%); median (Q1, Q3).

c Pearson's *X*^2^: Rao & Scott adjustment; design-based KruskalWallis test.

Among the 793 CHD patients, the majority did not experience all-cause mortality during follow-up, while approximately one-quarter experienced all-cause mortality. Hypertension was present in most participants. Patients who experienced all-cause mortality were significantly older, had lower income-to-poverty ratios, and consumed less alcohol compared to those who did not experience all-cause mortality (Table 1).

### 3.2 Distribution of heavy metals in blood

**Figure 1:**
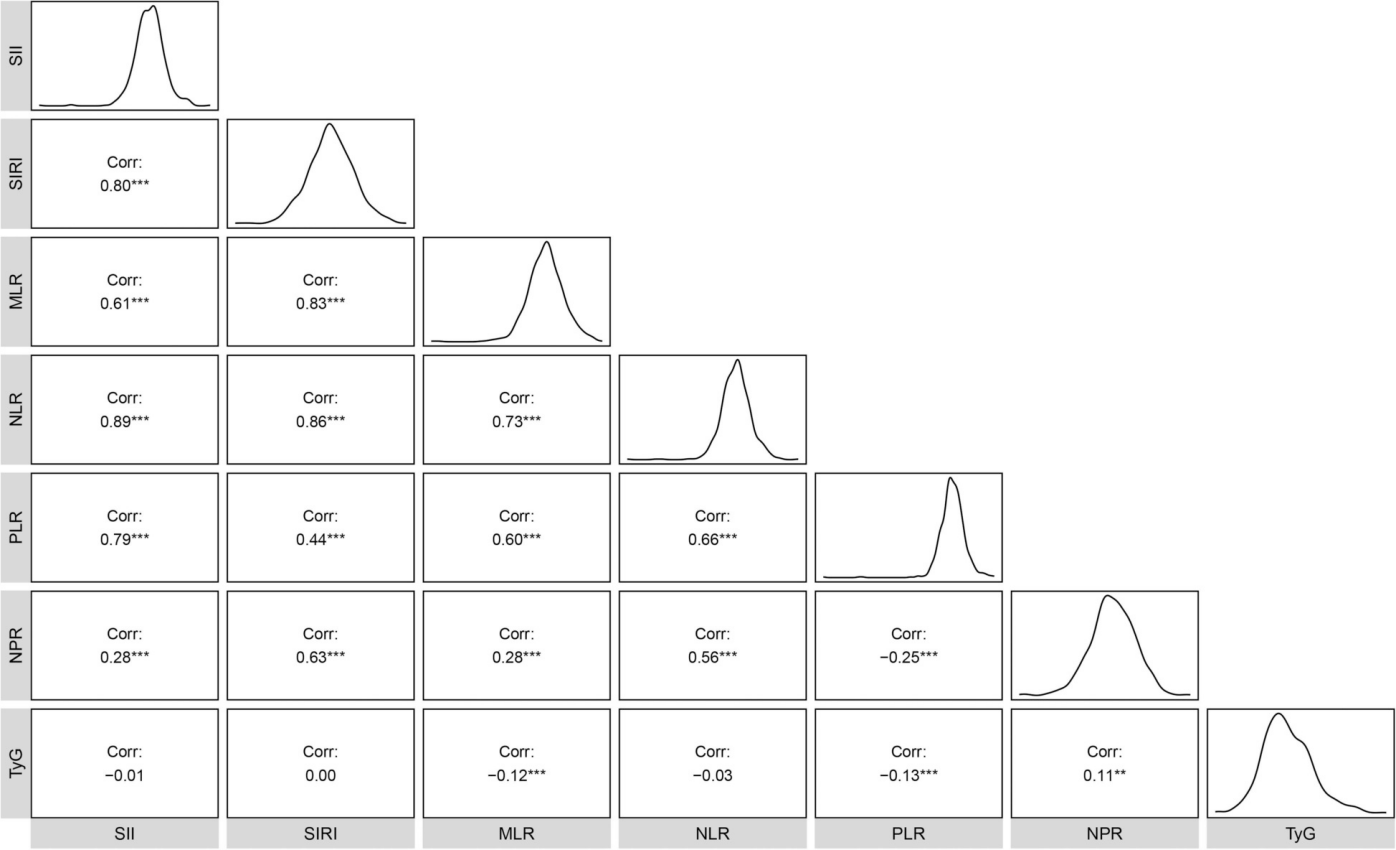
Correlations among TyG index, complete blood count derived inflammatory markers.

The mean ln-transformed values of SII, SIRI, MLR, NLR, PLR, NPR, and TyG were 6.13, 0.24, −1.16, 0.81, 4.73, −3.93, and 8.83, respectively. Strong correlations (|*r*| > 0.3) were observed between inflammatory markers, particularly involving NLR (Figure 1).

### 3.3 Association of death in patients with CHD With the ln-transformed TyG index and inflammatory indicators derived from CBCs

Table 3 presents the association between the log-transformed TyG index, CBC-derived inflammatory indices, and the risk of all-cause mortality in patients with CHD.

**Table 3:** Relationship between TyG index, CBC derived inflammatory markers and all-cause death in all participants.

Characteristic		Model 1			Model 2			Model 3		
		OR	95%CI	*P*	OR	95%CI	*P*	OR	95%CI	*P*
Log (Lead)	Continuous	2.3	1.70, 3.10	<0.001	1.69	1.12, 2.53	0.014	1.6	1.09, 2.34	0.017
	Q1	—	—		—	—		—	—	
	Q2	1.41	0.64, 3.11	0.38	0.85	0.38, 1.91	0.68	0.92	0.39, 2.15	0.834
	Q3	2.81	1.36, 5.81	0.006	1.53	0.73, 3.21	0.252	1.55	0.69, 3.46	0.274
	Q4	4.72	2.61, 8.54	<0.001	2.21	1.14, 4.31	0.021	2.12	1.08, 4.19	0.032
	P for trend	<0.001			0.005			0.007		
		Model 1			Model 2			Model 3		
		OR	95%CI	*P*	OR	95%CI	*P*	OR	95%CI	*P*
log(cadmium)	Continuous	1.54	1.27, 1.87	<0.001	1.52	1.17, 1.97	0.003	1.3	0.97, 1.75	0.08
	Q1	—	—		—	—		—	—	
	Q2	1.86	1.08, 3.20	0.025	1.53	0.79, 2.99	0.202	1.4	0.65, 3.00	0.369
	Q3	2.97	1.72, 5.14	<0.001	1.74	0.95, 3.21	0.074	1.54	0.78, 3.03	0.204
	Q4	2.74	1.53, 4.93	0.001	2.23	1.09, 4.58	0.03	1.61	0.70, 3.73	0.248
	P for trend	<0.001			0.02			0.203		
		Model 1			Model 2			Model 3		
		OR	95%CI	*P*	OR	95%CI	*P*	OR	95%CI	*P*
log(mercury)	Continuous	0.87	0.73, 1.04	0.115	0.97	0.78, 1.19	0.739	1.02	0.81, 1.27	0.889
	Q1	—	—		—	—		—	—	
	Q2	1.05	0.59, 1.90	0.856	1.3	0.65, 2.60	0.451	1.45	0.71, 2.95	0.292
	Q3	0.57	0.34, 0.97	0.039	0.64	0.35, 1.17	0.141	0.75	0.38, 1.48	0.386
	Q4	0.65	0.42, 1.02	0.061	0.84	0.49, 1.44	0.517	0.92	0.54, 1.56	0.74
	P for trend	0.011			0.147			0.337		
		Model 1			Model 2			Model 3		
		OR	95%CI	*P*	OR	95%CI	*P*	OR	95%CI	*P*
log(selenium)	Continuous	0.06	0.02, 0.27	<0.001	0.16	0.03, 0.96	0.046	0.17	0.03, 1.11	0.063
	Q1	—	—		—	—		—	—	
	Q2	0.57	0.35, 0.91	0.019	0.57	0.35, 0.95	0.031	0.58	0.34, 0.99	0.046
	Q3	0.45	0.27, 0.76	0.004	0.5	0.28, 0.89	0.019	0.46	0.24, 0.88	0.022
	Q4	0.52	0.33, 0.81	0.005	0.68	0.37, 1.24	0.203	0.72	0.38, 1.34	0.282
	P for trend	0.004			0.166			0.216		
		Model 1			Model 2			Model 3		
		OR	95%CI	*P*	OR	95%CI	*P*	OR	95%CI	*P*
log(manganese)	Continuous	0.42	0.24, 0.71	0.002	0.63	0.38, 1.05	0.074	0.68	0.42, 1.09	0.103
	Q1	—	—		—	—		—	—	
	Q2	0.56	0.33, 0.95	0.033	0.58	0.33, 1.03	0.063	0.63	0.33, 1.21	0.158
	Q3	0.57	0.36, 0.92	0.023	0.66	0.39, 1.13	0.126	0.78	0.44, 1.36	0.36
	Q4	0.49	0.27, 0.86	0.014	0.66	0.36, 1.24	0.193	0.69	0.36, 1.32	0.249
	P for trend	0.012			0.189			0.288		

Model 1 is not adjusted by any covariate; Model 2 adjusts gender, age, body mass index, race, education level, family income and poverty ratio on the basis of model 1; Model 3 adjusted smoking status, drinking status, hypertension, cholesterol, diabetes and physical activity on the basis of model 2.

Table 3 Correlation of Mortality in Patients With CHD With the ln-Transformed TyG Index and Inflammatory Indicators Derived from CBCs.

Table 3 presents the association between the log-transformed TyG index with CBC-derived inflammatory indices and the risk of all-cause mortality in patients with CHD. Model 1 was unadjusted for any covariates; model 2 was adjusted for gender, age, BMI, race, education, and household income relative to poverty based on model 1; and model 3 was adjusted for smoking status, alcohol consumption, hypertension, cholesterol, physical activity, and the presence of diabetes mellitus on the basis of model 2. The results indicated that in the unadjusted model1, SII, SIRI, MLR, NLR, and NPR showed significant positive associations with all-cause mortality risk (all *P* < 0.05), while PLR and TyG index did not demonstrate significant associations. These relationships persisted following comprehensive adjustment for confounding variables. Conversely, PLR and TyG did not exhibit significant correlations (*P* > 0.05) with the risk of all-cause mortality in this patients population. These relationships persisted following comprehensive adjustment for confounding variables. We converted the continuous TyG index, derived from a CBC inflammatory index, into a categorical variable. In Model1, which was unadjusted for covariates, we observed that participants in the fourth quartile exhibited a higher risk of all-cause mortality compared to those in the first quartile: SIRI (OR = 2.17, 95% CI = 1.17∼4.03, *P* = 0.015), MLR (OR = 2.47, 95% CI = 1.36∼4.46, *P* = 0.003), NLR (OR = 2.92, 95%CI = 1.49∼ 5.75, *P* = 0.002), and NPR (OR = 2.93, 95%CI = 1.51∼ 5.70, *P* = 0.002), all significantly associated with all-cause mortality (*P* < 0.05). Conversely, PLR (OR = 1.87, 95%CI = 0.93∼ 3.73, *P* = 0.076), SIRI (OR = 1.85, 95%CI = 0.92∼ 3.71, *P* = 0.083), and TyG (OR = 1.22, 95%CI = 0.60∼ 2.46, *P* = 0.576) showed no significant correlation with all-cause mortality (*P* > 0.05) (Table 3).

Table 4 presents the association between the TyG index and CBC-derived inflammatory indices, as well as the risk of cardiovascular mortality in patients with CAD. The results indicated that, without covariate adjustment, ln(SII) (OR = 2.16, 95% CI = 1.25∼3.72, *P* = 0.006), ln(SIRI) (OR = 2.34, 95% CI = 1.43∼3.83, *P* = 0.001), ln(MLR) (OR = 3.19, 95% CI = 1.65∼6.18, p\u003C0.001), ln(NLR) (OR = 2.96, 95% CI = 1.54∼ 5.69, *p* = 0.002), ln(PLR) (OR = 2.18, 95% CI = 1.19∼ 3.98, *P* = 0.012), and ln(NPR) (OR = 2.29, 95% CI = 1.03∼ 5.10, *P* = 0.042) were significantly correlated with the risk of cardiovascular mortality in patients with CAD (*P* < 0.05); however, the TyG index did not demonstrate a significant association with the risk of cardiovascular mortality in this population (*P* > 0.05). Upon comprehensive adjustment for confounders, ln(SII), ln(SIRI), ln(MLR), ln(NLR), and ln(PLR) were significantly associated with the risk of all-cause mortality in patients with CAD (*P*\u003C0.05). Conversely, ln(NPR) (OR = 2.01, 95% CI = 0.87∼ 4.65, *P* = 0.099) and TyG (OR = 1.3, 95% CI = 0.84∼ 2.02, *P* = 0.229) did not demonstrate significant associations with the risk of cardiovascular death in this patient population (*P* > 0.05). We transformed the continuous TyG index, derived from a CBC inflammatory index, into a categorical variable. In the absence of covariate adjustments, we observed that compared to participants in the first quartile, participants in the fourth quartile ln(SII) (OR = 3.08, 95% CI = 1.35∼7.04, *P* = 0.008), ln(MLR) (OR = 2.78, 95% CI = 1.17∼ 6.62, *P* = 0.022), ln(NLR) (OR = 3.12, 95%CI = 1.19∼ 8.20, *P* = 0.021), and ln(NPR) (OR = 3.67, 95%CI = 1.41∼ 9.58, *P* = 0.009) patients with CHD had an elevated risk of cardiovascular and cerebrovascular disease mortality and all were significantly associated with the risk of all-cause mortality were significantly correlated (*P* \u003C0.05). Conversely, ln(SIRI) (OR = 2.17, 95%CI = 0.90∼ 5.21, *P* = 0.083), ln(PLR) (OR = 1.64, 95%CI = 0.82∼ 3.26, *P* = 0.156), and TyG (OR = 1.29, 95%CI = 0.61∼ 2.74, *P* = 0.499) were not significantly correlated with the risk of cardiovascular and cerebrovascular mortality (*P* > 0.499). There was no significant association (*P* > 0.05). Upon complete adjustment for covariates, the fourth quartile of ln(SII), ln(NLR), and ln(NPR) were significantly associated with cardiovascular disease mortality compared with participants in the first quartile (*P* < 0.05), and the remaining were not significantly associated (*P* > 0.05) (Table 4).

**Table 4:** Relationship between TyG index, CBC derived inflammatory markers and cerebrovascular diseases in all.

Characteristic		Model 1			Model 2			Model 3		
		OR	95%CI	*P*	OR	95%CI	*P*	OR	95%CI	*P*
log (Lead)	Continuous	2.06	1.50, 2.85	<0.001	1.47	0.97, 2.23	0.068	1.47	1.06, 2.02	0.021
	Q1	—	—		—	—		—	—	
	Q2	2.1	0.61, 7.27	0.236	1.38	0.39, 4.87	0.607	1.42	0.41, 4.93	0.566
	Q3	3.64	1.37, 9.65	0.011	2.1	0.79, 5.61	0.133	2.17	0.86, 5.49	0.096
	Q4	5.45	1.68, 17.7	0.006	2.5	0.68, 9.15	0.161	2.48	0.75, 8.22	0.13
	P for trend	<0.001			0.06			0.044		
		Model 1			Model 2			Model 3		
		OR	95%CI	*P*	OR	95%CI	*P*	OR	95%CI	*P*
log(cadmium)	Continuous	1.28	0.96, 1.71	0.091	1.18	0.80, 1.74	0.395	1.09	0.67, 1.78	0.707
	Q1	—	—		—	—		—	—	
	Q2	2.02	0.96, 4.25	0.062	1.57	0.66, 3.69	0.296	1.61	0.64, 4.04	0.292
	Q3	2.12	0.94, 4.82	0.071	1.04	0.43, 2.52	0.924	1.04	0.41, 2.69	0.925
	Q4	1.83	0.73, 4.61	0.195	1.32	0.47, 3.73	0.59	1.19	0.32, 4.36	0.784
	P for trend	0.169			0.824			0.999		
		Model 1			Model 2			Model 3		
		OR	95%CI	*P*	OR	95%CI	*P*	OR	95%CI	*P*
log(mercury)	Continuous	0.74	0.47, 1.17	0.197	0.84	0.52, 1.37	0.48	0.88	0.53, 1.46	0.604
	Q1	—	—		—	—		—	—	
	Q2	0.78	0.29, 2.10	0.612	0.85	0.29, 2.51	0.765	0.85	0.27, 2.72	0.773
	Q3	0.31	0.15, 0.65	0.002	0.33	0.15, 0.71	0.006	0.35	0.15, 0.80	0.015
	Q4	0.49	0.20, 1.21	0.119	0.67	0.25, 1.80	0.41	0.72	0.25, 2.13	0.54
	P for trend	0.036			0.129			0.225		
		Model 1			Model 2			Model 3		
		OR	95%CI	*P*	OR	95%CI	*P*	OR	95%CI	*P*
log(selenium)	Continuous	0.09	0.01, 0.75	0.027	0.34	0.04, 2.80	0.305	0.29	0.03, 2.78	0.268
	Q1	—	—		—	—		—	—	
	Q2	0.65	0.35, 1.19	0.156	0.7	0.39, 1.26	0.228	0.64	0.35, 1.16	0.133
	Q3	0.32	0.16, 0.63	0.002	0.4	0.19, 0.83	0.015	0.35	0.16, 0.76	0.011
	Q4	0.6	0.28, 1.26	0.173	0.89	0.38, 2.06	0.775	0.82	0.34, 1.99	0.649
	P for trend	0.105			0.541			0.456		
		Model 1			Model 2			Model 3		
		OR	95%CI	*P*	OR	95%CI	*P*	OR	95%CI	*P*
log(manganese)	Continuous	0.4	0.17, 0.92	0.032	0.65	0.28, 1.50	0.304	0.73	0.30, 1.77	0.467
	Q1	—	—		—	—		—	—	
	Q2	0.38	0.16, 0.92	0.033	0.4	0.14, 1.17	0.092	0.38	0.12, 1.21	0.096
	Q3	0.46	0.26, 0.83	0.011	0.58	0.28, 1.19	0.131	0.61	0.27, 1.36	0.213
	Q4	0.44	0.24, 0.81	0.01	0.62	0.30, 1.27	0.18	0.65	0.31, 1.37	0.244
	P for trend	0.017			0.225			0.324		

Model 1 is not adjusted by any covariate; Model 2 adjusts gender, age, body mass index, race, education level, family income and poverty ratio on the basis of model 1; Model 3 adjusted smoking status, drinking status, hypertension, cholesterol, diabetes and physical activity on the basis of model 2.

### 3.4 Dose-response of TyG index vs. CBC-derived inflammatory indices and risk of all-cause and cardiovascular disease mortality in patients with coronary artery disease

Regression modeling based on restricted cubic spline (RCS) to analyze the dose-response relationship between the TyG index and inflammatory indicators generated from CBC concerning the risk of all-cause mortality and cardiovascular disease mortality in patients with CHD.

**Figure 2:**
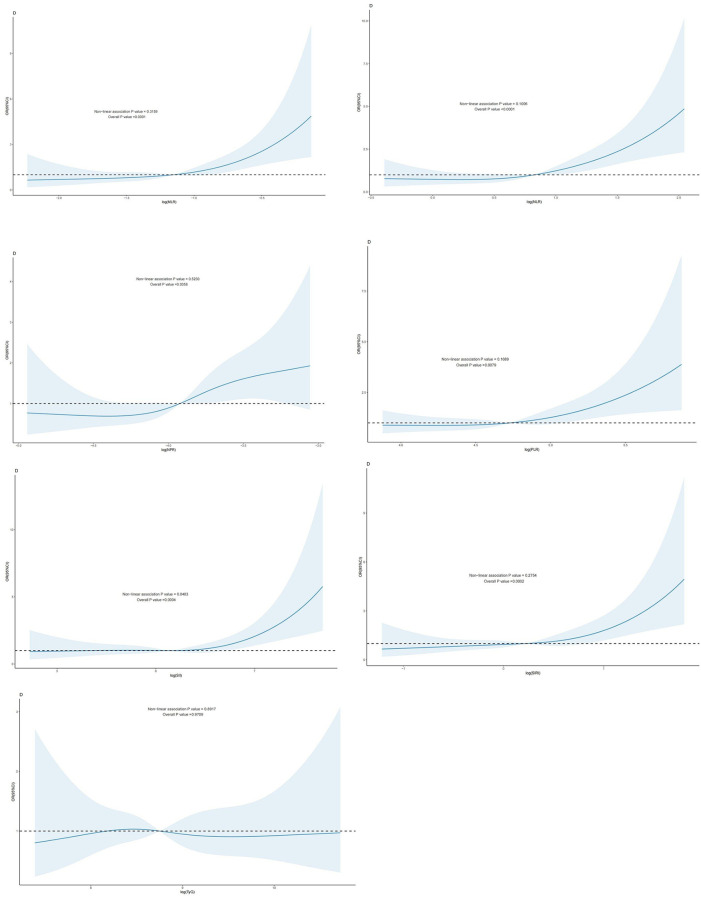
Dose-response effect between blood TyG index and CBC-derived inflammation index and risk of all-cause mortality in patients with CHD. Relationship between the dose response between blood MLR and risk of all-cause mortality in patients with CHD (fully adjusted RCS model), with the baseline value (*β* = 1) indicated by a dashed line and the inflection point occurring at −1.82 (after logarithmic transformation). **(B)**: relationship between the dose response between NLR in blood and the risk of all-cause mortality in patients with coronary artery disease (fully adjusted RCS model), with the baseline value (*β* = 1) indicated by a dashed line and the inflection point occurring at 0.05 (after logarithmic transformation) **(C)**: relationship between the dose response between NPR in blood and the risk of all-cause mortality in patients with coronary artery disease is linear (fully adjusted RCS model). **(D)**: the relationship between the dose-response between PLR in blood and the risk of all-cause mortality in patients with CHD (fully adjusted RCS model), with the baseline value (*β* = 1) indicated by a dashed line and the inflection point occurring at 4.46 (after logarithmic transformation). **(E)** Dose-response relationship between SII in blood and risk of all-cause mortality in patients with coronary artery disease (fully adjusted RCS model), with baseline value (*β* = 1) indicated by a dashed line and inflection point occurring at 5.4 (log-transformed). **(F)** Relationship between dose-response between SIRI in blood and risk of all-cause mortality in patients with coronary artery disease (fully adjusted RCS model), with baseline value (*β* = 1) indicated by a dashed line and inflection point occurring at 0.23 (log-transformed). **(G)**: the relationship between the dose-response between blood TyG and the risk of all-cause mortality in patients with CHD was linear (fully adjusted RCS model). The model was adjusted to account for the following variables: age, gender, race, education level, exercise, smoking status, BMI, household PIR, drinking status, education, hyperlipidemia, and hypertension.

For all-cause mortality, ln(NPR) and TyG exhibited a linear dose-response relationship in patients with CAD (*P* -nonlinear value > 0.05), whereas ln(SII), ln(SIRI), ln(MLR), ln(NLR), and ln(PLR) demonstrated a nonlinear dose-response relationship in this patient population (*P* -nonlinear value < 0.05). The inflection points of ln(SII), ln(SIRI), ln(MLR), ln(NLR), and ln(PLR) for the risk of all-cause mortality in individuals with CAD were 5.4, 0.23, −1.82, 0.05, and 4.46, respectively (Figure 2).

**Figure 3:**
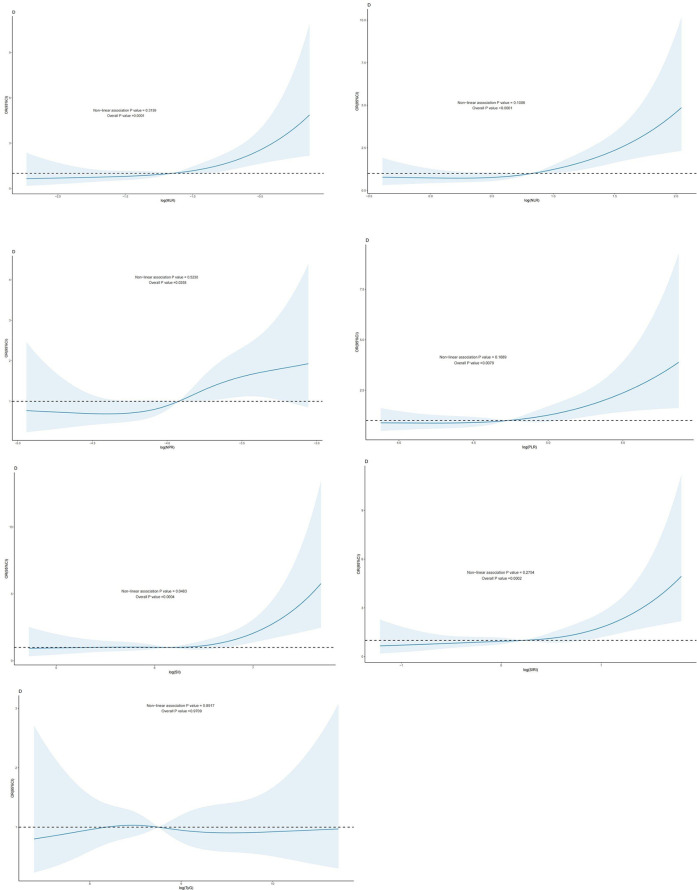
Dose-response effect between blood TyG index and CBC-derived inflammatory indices and risk of cardiovascular death in patients with coronary artery disease: the relationship between the dose-response between blood MLR and the risk of cardiovascular death in patients with coronary artery disease is linear (fully adjusted RCS model) **(B)**: the relationship between the dose-response between blood NLR and the risk of cardiovascular death in patients with coronary artery disease is linear (fully adjusted RCS model) **(C)**: the relationship between blood NPR and the risk of cardiovascular death in patients with coronary artery disease is linear (fully adjusted RCS model). relationship is linear (fully adjusted RCS model). **(D)**: the relationship between the dose-response between PLR in blood and the risk of cardiovascular death in patients with CHD is linear (fully adjusted RCS model) **(E)** The relationship between the dose-response between SII in blood and the risk of all-cause mortality in patients with CHD (fully adjusted RCS model), with the baseline value (*β* = 1) indicated by a dashed line and the inflection point occurring at 5.36 (after logarithmic transformation). **(F)** The relationship between the dose-response between SIRI in blood and the risk of cardiovascular death in patients with coronary artery disease is linear (fully adjusted RCS model). **(G)**: The relationship between the dose-response between TyG in blood and the risk of cardiovascular death in patients with coronary artery disease is linear (fully adjusted RCS model). The model was adjusted to take into account the following variables: age, gender, race, education level, exercise, smoking status, BMI, household PIR, drinking status, education level, hyperlipidemia, and hypertension.

In individuals with CAD, a nonlinear relationship was seen between the log(SII) dosage response and the risk of cardiovascular mortality (*P*-nonlinear value < 0.05). ln(SIRI), ln(MLR), ln(NLR), ln(PLR), ln(NPR), TyG, and the risk of cardiovascular mortality in patients with CAD exhibited a linear correlation (*P*- nonlinear value > 0.05). A linear relationship existed when the *P* -nonlinear value exceeded 0.05 (Figure 3).

WQS Regression Analysis of the TyG Index and CBC-Derived Inflammatory Indicators in Relation to Mortality in patients with CHD.

**Figure 4:**
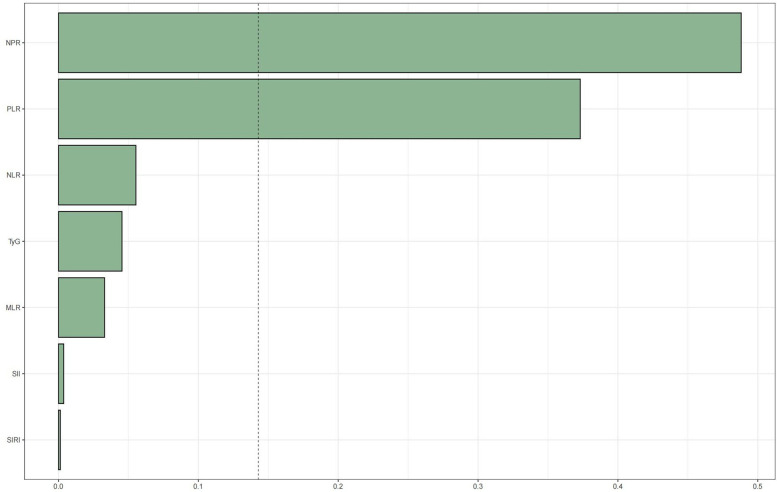
WQS regression analysis of TyG index vs. CBC-derived inflammatory markers and all-cause mortality in patients with CHD. The model was adjusted taking into account the following variables: age, gender, race, education level, exercise, smoking status, BMI, household PIR, drinking status, education, hyperlipidemia, and hypertension.

**Figure 5:**
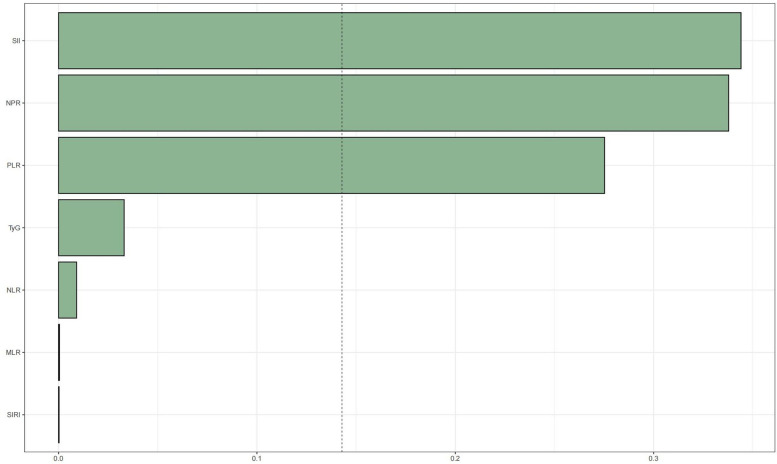
WQS regression analysis of TyG index vs. CBC-derived inflammatory markers and cardiovascular mortality in patients with CHD.The model was adjusted taking into account the following variables: age, gender, race, education level, exercise, smoking status, BMI, household PIR, drinking status, education level, hyperlipidemia, and hypertension.

WQS regression was employed to evaluate the cumulative impact of the TyG index and the inflammation index derived from CBC on mortality risk in patients with CAD. As illustrated in Figures 4, 5, the TyG index and the WQS index of inflammation derived from CBC were significantly and positively correlated with the risk of all-cause mortality in patients with CAD [OR: 1.61, 95% CI (1.13, 2.29), *P* = 0.009]. Notably, NPR (48.82%) was the predominant contributor to this association. The relationship between the TyG index and the WQS index, an inflammation marker derived from CBC, was significantly and positively correlated with the risk of all-cause mortality in patients with CAD. This association was particularly pronounced for cardiovascular disease mortality [OR: 2.2, 95% CI (1.42, 3.42), *P* < 0.001], with the SII (34.4%) demonstrating the strongest positive association between the TyG index and the WQS index, indicating the greatest weight of association with of cardiovascular death risk in patients with CAD (Figures 4, 5).

## 4 Discussion

The risk of all-cause mortality in patients with CAD was substantially correlated with SII, SIRI, MLR, NLR, and NPR. Upon adjusting for various confounding variables, the indicators remained strongly associated with the probability of all-cause mortality. SII, SIRI, MLR, NLR, and PLR were associated with the risk of cardiovascular mortality. The TyG index and inflammatory indicators obtained from CBC were strongly and positively correlated with the risk of all-cause mortality and cardiovascular death in patients with CAD, with NPR and SII exerting the greatest impact. The results emphasize the predictive significance of NPR and SII concerning mortality risk in patients with CAD, indicating that decreasing NPR and SII levels through intervention in the inflammatory state may be crucial for enhancing patient prognosis.

CHD is a common cardiovascular disease in clinical practice and a significant cause of morbidity and mortality worldwide. According to NHANES data from 2017 to 2020, approximately 20.5 million patients in the United States are affected by CHD, and the prevalence is significantly higher in men than in women [16]. CHD accounts for the highest proportion of heart disease deaths in the United States, with an average of 1 in 6 deaths caused by CHD [17]. In addition, medical expenditures related to CHD continue to climb in developed countries, placing a heavy burden on the healthcare system. The pathogenesis of CHD is due to the continuous accumulation of fat or harmful cholesterol in the arterial wall, which ultimately leads to arterial wall narrowing and obstruction [18]. The development and progression of coronary atherosclerosis can be facilitated by factors such as vascular endothelial damage, lipid metabolism disorders, and inflammatory responses, which in turn lead to coronary heart disease [19, 20]. In recent years, the TyG index, an indicator of IR, and CBC-derived inflammatory indices have gradually garnered attention.

The Systemic Immune Inflammatory Index (SII) is an innovative biomarker that objectively evaluates the inflammatory and immunological status of a patient, incorporating additional inflammatory and immune signals that indicate the body's condition. The SII was originally utilized as a novel inflammatory marker for malignancy-associated conditions (21) and has subsequently been broadened to encompass various non-oncological diseases, including peripheral arterial disease, cerebral hemorrhage, diabetic nephropathy, non-obstructive CAD, and acute myocardial infarction. Previous studies have indicated that SII correlates positively with an elevated risk of major adverse cardiovascular events in individuals with CAD (22). Our study further revealed that SII was significantly positively correlated with both all-cause and cardiovascular deaths in patients with CAD, with SII being the most substantial contributor to the risk of cardiovascular mortality among all inflammatory markers. This may assist in the early identification of high-risk groups for all-cause and cardiovascular mortality in patients with CAD, as well as in the more precise assessment of disease severity and patients prognosis.

IR denotes the diminished physiological efficacy of insulin in the body, impairing its capacity to metabolize glucose. IR leads to metabolic abnormalities such as dyslipidemia, hypertension, and hyperglycemia, which are risk factors for CVD. The TyG index was initially employed to evaluate IR in patients with type 2 diabetes mellitus. It serves as a dependable alternative to the high insulin clamp test, calculated as ln [fasting glucose × fasting triglycerides/2]. Furthermore, the TyG index has demonstrated superior predictive capability in identifying diabetes patients compared to fasting glucose and triglycerides alone. Initially, research on the TyG index focused on the endocrine field. However, its cardiovascular significance recently has gained increasing attention. Studies have demonstrated that the TyG index is associated with an elevated risk of adverse cardiovascular outcomes, including coronary artery calcification, carotid atherosclerosis, and heart failure. Furthermore, it is an independent risk factor for CAD development (23). Supporting this, a large 2024 Korean cohort study found that higher TyG index levels were independently associated with elevated risks of all-cause mortality and cardiovascular death (24), Similarly, a Chinese single-center prospective cohort study demonstrated that the TyG index in patients with CHD during hospitalization was nonlinearly correlated with all-cause mortality and cardiovascular MACE events. Specifically, a TyG index exceeding 8.77 and 8.62, respectively, was significantly associated with an increased risk of all-cause mortality and adverse cardiovascular events.

Our investigation could not identify a significant correlation between the TyG index and the risk of all-cause mortality or cardiovascular mortality in a specific cohort of patients with CHD. This finding may be ascribed to the focus of our investigation on a specific patient population with CAD, where multiple confounding factors, including age, gender, smoking history, hypertension, and dyslipidemia, may have obscured the impact of the TyG index. The association between the TyG index and mortality risk in individuals with CHD may not be linear and could be influenced by additional biological mechanisms and mediators. Our findings emphasize the necessity of evaluating the predictive significance of the TyG index across diverse patient populations and indicate that subsequent research should further investigate the interplay between the TyG index and other risk factors, as well as its targeted application in the management of CHD. Consequently, although our findings contradict certain aspects of the existing literature, they furnish novel insights into the relevance of the TyG index in patients with CHD and present valuable guidance for future research endeavors.

Our study also has several drawbacks. Firstly, hematological indices were assessed in a single-point sample, which may fluctuate over a brief duration, perhaps failing to adequately reflect an individual's normal exposure. Secondly, confounding influences arising from psychosocial variables, genetic predisposition, unidentified confounders, or random variation cannot be excluded in this study. Ultimately, additional experimental investigations are required to elucidate the mechanism of the TyG index in comparison to inflammatory indices obtained from CBC concerning the risk of all-cause and cardiovascular disease mortality in patients with CAD.

## 5 Conclusion

In conclusion, the incorporation of the WQS model in our study demonstrated that the TyG index and CBC-derived inflammatory indicators are potent influencers of the risk of all-cause and cardiovascular mortality in patients with CAD. Furthermore, the combination of these indicators may be advantageous in mitigating premature mortality in this patient population.

Clinical Implications and Future Directions: These findings have several important clinical applications. First, CBC-derived inflammatory indices, particularly NPR and SII, could be incorporated into routine risk stratification protocols for CHD patients, as they are readily available from standard laboratory tests. Second, serial monitoring of these biomarkers may help identify patients at highest risk for adverse outcomes, enabling more targeted interventions. Third, future research should investigate whether anti-inflammatory treatments guided by these markers can improve patient outcomes.

Healthcare providers should consider implementing these biomarkers in clinical decision-making algorithms to enhance prognostic accuracy beyond traditional risk factors. The ease of obtaining these markers from routine blood tests makes them particularly suitable for widespread clinical application.

## Data Availability

The raw data supporting the conclusions of this article will be made available by the authors, without undue reservation.
