# Correction: TyG Index and CBC-derived inflammatory indicators individual and mixed effects on all-cause and cardiovascular disease deaths in patients with CHD

**DOI:** 10.3389/fcvm.2025.1703683

**Published:** 2025-11-06

**Authors:** Lipeng Cai, Jiangrong Yan, Lei Sun, Weichao Dan

**Affiliations:** 1 Department of Cardiology, The Third People’s Hospital of Huizhou, The Affiliated Hospital of Guangzhou Medical University, Huizhou, Guangdong, China; 2 Cardiovascular Department, Huizhou Affiliated Hospital of Sun Yat-sen University, Huizhou, Guangdong, China; 3 Orthopedic Department, Guoyao North Hospital, Baotou, Inner Mongolia Autonomous Region, China

**Keywords:** TyG index, CBC inflammatory marker, all-cause mortality, cardiovascular mortality, environmental health, CBC-derived inflammatory index, CHD, cardiovascular disease mortality

There was a mistake in the caption of Table 3 and Table 4 as published. The Table 3 caption was erroneously written as “Relationship between blood heavy metals and all-cause death in all participants” and the Table 4 caption was erroneously written as “Relationship between blood heavy metals and cardiovascular death in all participants”. The corrected captions of Table 3 and Table 4 appear below.

“Table 3 Relationship between TyG index, CBC derived inflammatory markers and all-cause death in all participants.”

“Table 4 Relationship between TyG index, CBC derived inflammatory markers and cerebrovascular diseases in all.”

The content about heavy metals (third paragraph of the Discussion section) has been removed. The first sentence of the third paragraph has been retained as the last sentence of the second paragraph.

A correction has been made to the section Discussion, paragraph 2:

“CHD is a common cardiovascular disease in clinical practice and a significant cause of morbidity and mortality worldwide. According to NHANES data from 2017 to 2020, approximately 20.5 million patients in the United States are affected by CHD, and the prevalence is significantly higher in men than in women [16]. CHD accounts for the highest proportion of heart disease deaths in the United States, with an average of 1 in 6 deaths caused by CHD [17]. In addition, medical expenditures related to CHD continue to climb in developed countries, placing a heavy burden on the healthcare system. The pathogenesis of CHD is due to the continuous accumulation of fat or harmful cholesterol in the arterial wall, which ultimately leads to arterial wall narrowing and obstruction [18]. The development and progression of coronary atherosclerosis can be facilitated by factors such as vascular endothelial damage, lipid metabolism disorders, and inflammatory responses, which in turn lead to coronary heart disease [19, 20]. In recent years, the TyG index, an indicator of IR, and CBC-derived inflammatory indices have gradually garnered attention.”

The original version of this article has been updated.

